# Cell Cycle-Associated CXCR4 Expression in Germinal Center B Cells and Its Implications on Affinity Maturation

**DOI:** 10.3389/fimmu.2018.01313

**Published:** 2018-06-12

**Authors:** Tom S. Weber

**Affiliations:** ^1^Molecular Medicine Division, Walter and Eliza Hall Institute for Medical Research, Parkville, VIC, Australia; ^2^Department of Medical Biology, The University of Melbourne, Parkville, VIC, Australia

**Keywords:** germinal center, cell cycle, BrdU, CXCR4, CXCL12, dark zone, light zone, affinity maturation

## Abstract

Adaptation of antibody-mediated immunity occurs in germinal centers (GC). It is where affinity maturation, class switching, memory and plasma cell differentiation synergize to generate specific high-affinity antibodies that aid both to clear and protect against reinfection of invading pathogens. Within GCs, light and dark zone are two compartments instrumental in regulating this process, by segregating T cell-dependent selection and differentiation from generation of GC B cells bearing hypermutated antigen receptors. Spatial segregation of GC B cells into the two zones relies on the chemokine receptor CXCR4, with textbooks attributing high and low expression to a dark and light zone phenotype. Interestingly, this bipolarity is not reflected in the CXCR4 expression profile of GC B cells, which is highly variable and unimodal, indicating a continuum of intermediate CXCR4 levels rather than a binary dark or light zone phenotype. Here, analysis of published BrdU pulse-chase data reveals that throughout cell cycle, average CXCR4 expression in GC B cells steadily increases close to twofold, scaling with cell surface area. CXCR4 expression in recently divided GC B cells in G0/G1 or early S phase shows intermediate levels compared to cells in G2M phase, consistent with their smaller size. The lowest number of CXCR4 receptors are displayed by relatively aged GC B cells in G0/G1 or early S phase. The latter, upon progressing through S phase, however, ramp up relative CXCR4 expression twice as much as recently divided cells. Twelve hours after the BrdU pulse, labeled GC B cells, while initially in S phase, are desynchronized in terms of cell cycle and match the CXCR4 profile of unlabeled cells. A model is discussed in which CXCR4 expression in GC B cell increases with cell cycle and cell surface area, with highest levels in G2 and M phase, coinciding with GC B cell receptor signaling in G2 and immediately preceding activation-induced cytidine deaminase (AID) activity in early G1. In the model, GC B cells compete for CXCL12 expression on the basis of their CXCR4 expression, gaining a relative advantage as they progress in cell cycle, but loosing the advantage at the moment they divide.

## 1 Introduction

Germinal centers (GC) play a fundamental role in adaptive humoral immunity by providing the niche in which antigen-specific activated B cells undergo class switching, affinity maturation, memory, and plasma cell differentiation (1–3). GCs develop in secondary lymphoid organs a few days post-immunization or infection. Founded by 20–200 activated B cell clones each (4, 5), they exponentially grow in size, to form a relatively stable broadly sized population (6) and wane several weeks post-immunization or after the infection is cleared.

Mature GCs contain GC B cells, T helper cells, tingible body macrophages, a network of follicular dendritic (7), and CXCL12-expressing reticular cells (8). Each cell type is assigned a specific function in what is collectively termed the germinal center reaction. B cells, as potential effector cells, play a chief part. They generate large amounts of progeny with altered B cell receptors via intense proliferation and activation-induced cytidine deaminase (AID)-dependent somatic hypermutation (9, 10). Some of the progeny undergo AID-dependent class switching (11) and/or division-linked differentiation into memory (12) and long-lived plasma cells (13, 14), while others undergo apoptosis (15). Most memory cells are derived early (16) while plasma cells are generated late in the response (17). Key in this complex cell fate decision program are T helper cells that provide survival signals to higher affinity GC B cell variants at the expense of lower affinity peers (3, 18–21). Tingible body macrophages engulf apoptotic GC B cells and debris through phagocytosis and have been proposed to play a role in downregulating the GC reaction (22). Follicular dendritic cells stock and supply opsonized antigen coated on their surface via the Fc-receptor (23–25). Finally, reticular cells produce CXCL12 (8, 26), the ligand for CXCR4, a chemokine receptor essential in polarizing GCs into the light zone (LZ) and dark zone (DZ) (27).

The DZ and LZ are two histologically well-defined regions within mature GCs (27–30). In the DZ, GC B cells divide more frequently (31), and AID, the enzyme required for somatic hypermutation and antibody class switching, is upregulated (32). In the LZ, follicular dendritic cell network carry and present antigen in form of iccosomes (33), while T helper cells (crucial for providing survival signals to GC B cells), apoptotic cells, and tingible body macrophages are more abundant. Taken together these and other observations have led to a model in which GC B cells bearing hypermutated and/or switched antigen receptors are generated primarily in the DZ, and antibody affinity-dependent selection of GC B cells is more likely to happen in the LZ (1, 3). Implicitly, this model assumes some degree of recycling between the DZ and LZ, the importance of which has long been a matter of debate (34–37).

A major advance in the understanding of cell migration within GCs came with the advent of two-photon live microscopy and live imaging (30, 38, 39). Monitoring GCs in lymph nodes of anesthetized mice shows highly motile GC B cell, crawling on FDC networks in the LZ, with frequent but mostly short interactions between B and T cells (21, 38, 39). Due to the limited imaging time windows, precise flux rates between the DZ and LZ have been challenging to infer with this experimental system (40, 41). To overcome this limitation and quantify cell migration from DZ to LZ over longer time frames an elegant experimental system was developed in which photoactivatable GFP expressing GC B cells were activated in situ in anesthetized mice in either dark or light zone and their position recorded several hours later (42). This confirmed substantial fluxes between the two zones (43), in line with theoretical predictions developed earlier (36, 37, 44).

The main outcome of the GC reaction is affinity maturation (45–47), the increase in average binding affinity of circulating antibodies. Typically described akin to Darwinian evolution (48–51), it involves rounds of proliferation and mutation of the genes coding for the B cell receptor variable region (predominantly in the DZ) followed by selection of higher affinity variants and clearance of GC B cells carrying non-functional or low affinity receptors (predominantly in the LZ). While the process by which B cells mutate their BCRs is relatively well understood at the molecular level (52, 53), the details regarding the selection process remain controversial (36, 54–56). Historically perceived as highly efficient (57), some studies including recent work based on a stochastic multicolor Aid-Cre reporter mice suggests that selection is less stringent than initially thought (5, 58, 59). Whether low stringency aids in maintaining polyclonality and hence antibody diversity (60), or represents the highest level achievable under biological conditions awaits to be elucidated. Irrespectively of the degree of selection pressure, however, consensus is that signals from T helper cells are the limiting factor for GC B cell survival (18, 19).

In this work, published BrdU pulse-chase data of GC B cells is reanalyzed (39). In a first section, proportions of pulse-labeled BrdU+ cells are tracked over time in order to infer turnover and survival rates of recently divided GC B cells. In section two, CXCR4 expression is compared between subpopulations that differ in their cell cycle position and DNA content. This reveals that GC B cell CXCR4 expression steadily increases throughout cell cycle. In two subsequent sections, pulse-chase data at additional time points after the BrdU pulse are analyzed. This leads to the identification of two distinct G0/G1 populations that differ in average CXCR4 expression: a first population that has recently divided with intermediate levels, and a second population that has not been in cycle for several hours with low levels. In the last section, analysis of data from cells harvested 12 h after the pulse shows BrdU labeled and unlabeled cells have converged at that time in terms of CXCR4 expression and cell cycle distribution.

## 2 Materials and Methods

### 2.1 Experimental Procedures

Immunization of mice, BrdU pulse chase, and staining procedures are detailed in the original study (39). In brief, B6 mice were immunized subcutaneously with (4-hydroxy-3-nitrophenyl)acetyl-chicken gamma globulin (NP30-CGG, Biosearch Technologies) emulsified in complete Freund’s adjuvant (Sigma-Aldrich) at 7 different sites. Two weeks later, BrdU (Sigma-Aldrich or BD Pharmingen) in PBS was administered by a single intraperitoneal injection and cells from a total of ten mice were harvested at 30 min, 2, 3.5, 5, 8, and 12 h after the pulse (Figure S1A in Supplementary Material). Draining lymph nodes were pooled for the analysis. BrdU and DNA content were determined using the FITC BrdU flow kit (BD Pharmingen). DAPI was added prior to FACS. For down-stream analysis, GC B cells were defined as CD4− CD19+ Fas+ IgDlow cells (Figure S1B in Supplementary Material).

### 2.2 Analysis of BrdU Pulse-Chase Data

Interpretation of pulse-chase data relies upon well-established relationships between cell cycle progression, cell division, and BrdU/DNA content. Figures S1C–F in Supplementary Material shows BrdU and DNA content for three hypothetical “cells” as they evolve over time after the pulse. For illustrative purposes, we assume a clockwise cell cycle progression with G0/G1, S1, S2, S3, S4, S5, G2M as discrete cell cycle states of identical duration (arbitrarily set to 1 h) and no death (Figure S1B in Supplementary Material). A more realistic model would take into account different phase duration, biologically variability in cell cycle progression and apoptosis (61).

In Figure S1 in Supplementary Material, initially one of the three cells is in mid-S phase, and has, therefore, incorporated BrdU (orange cell in Figure S1C in Supplementary Material). A second cell is in G0/G1 phase, and will enter S phase shortly after (red cell). A third cell is in G2M phase (blue cell). Both cells in G0/G1 and G2M are not labeled by BrdU as they are not synthesizing DNA at the time of the pulse. After 3 h (Figure S1D in Supplementary Material), the BrdU+ cell has reached G2M phase, the cell initially in G0/G1 phase has progressed to mid-S phase, and the cell initially in G2M has divided and its progeny are in early S phase. One hour later (Figure S1E in Supplementary Material), the BrdU+ cell has divided as well, and progeny are in G0/G1 phase. Their BrdU content is half compared to the mother cell and distinguishable from non-labeled cells. The three unlabeled cells have increased their DNA content further, and therefore have “moved” to the right along the DNA axis.

When comparing the schematics in Figures S1C–F in Supplementary Material to real data, there are several additional complexities that need to be considered: (a) initially cells are desynchronized, i.e., are distributed all over the cell cycle, (b) cell cycle phase durations are variable (e.g., G0/G1 is typically longer than G2M phase), (c) cell cycle progression has a significant stochastic component, (d) the same cells are not tracked over time, and (e) cells undergo apoptosis or differentiate. Despite these differences the simplified model (whose sole purpose is illustration) follows the same logic, and therefore serves to understand the underlying dynamics of real bi-variate BrdU-DNA content scatter plots after the BrdU pulse.

### 2.3 Statistical Analysis

Error bars in the graphs as well as confidence intervals reported corresponds to mean ± two SDs throughout the text. For statistical significance of the difference in means between two samples, the Welch two sample *t*-test was applied, with *p*-values <0.05 being regarded as statistically significant. For linear regression, *p*-values were computed using the *F*-test with null hypothesis of the slope being equal zero. Statistical significance between coefficient of variations was tested using the Feltz and Miller test (62) and implemented in the R package “cvequality.” All statistical tests were performed using the computing environment R.

## 3 Results

### 3.1 Pulse-Chasing GC B Cells Confirms High Turnover Rates and Reveals Survival Times Longer Than 5 h After Birth

**Figure 1:**
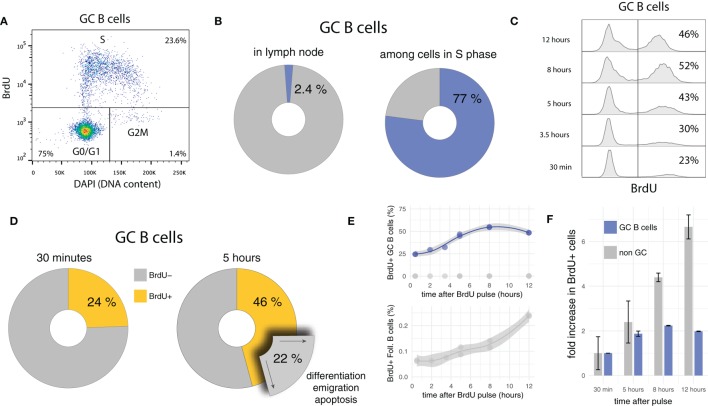
BrdU pulse chasing of GC B cells. **(A)** Bi-variate scatter plot of DAPI (DNA content) versus BrdU 30 min after the pulse identifies cells in G0/G1 phase, in S phase, and in G2M phase. **(B)** Only a small fraction of cells (2.4 **±** 1%) in draining lymph nodes are GC B cells, but due to their fast cycling, they dominate the population of cells in S phase (77 **±** 0.01%). **(C)** Representative BrdU pulse-chase data, in which GC B cells are harvested at different times (from 30 min up to 12 h) after the BrdU pulse. **(D)** Between 30 min and 5 h after the BrdU pulse, the proportion of BrdU+ among GC B cells increases in average by 22%. Assuming quasi-steady state (during 4.5 h), an equivalent proportion of GC B cells must egress from the GC through differentiation or apoptosis during the same period of time. **(E,F)** Proportions of BrdU+ GC B cells approximately double within 5–6 h, and then stabilize at approximately 50%, twofold of the initial value. By contrast, cells that do not express the GC B cell marker (e.g., follicular B cells) initially contain few BrdU+ cells, but continuously increase this proportion over time.

GC B cells are highly proliferative, a feature likely to be critical in keeping pace with rapidly evolving pathogens and/or in producing high-affinity antibodies most promptly after onset of infection. Consistent with this hypothesis and previous studies (26, 30, 54, 63, 64), in the present data, 24 ± 0.04% GC B cells (compared to 0.063 ± 0.006% among follicular B cells) incorporates the thymidine analog BrdU, which implies that about one in four GC B cells is replicating DNA at any time during the experiment. Although GC B cells are only a minority (2.4 ± 1%) in draining lymph nodes (LN), they represent 77 ± 0.01% of cells in synthesis (S) phase (Figures 1A,B).

Some quantitative and qualitative deductions in terms of proliferation and selection/differentiation can be made by analyzing the kinetics of BrdU pulse-labeled cells over time (pulse-chase). Because a single BrdU+ mother cell gives rise to two BrdU+ daughter cells, if the daughter cells survive and maintain a GC B cell phenotype after division, the proportion of BrdU+ cells has to increase, as soon as labeled cells in late S phase complete G2 and M phase and divide. If subsequent survival is longer than the duration of S phase, a doubling in the frequency is expected. Indeed, after a short initial delay, the proportion of BrdU+ GC B cells increases, and almost doubles from 24 ± 0.04 to 46 ± 2% within the first 5 h (Figures 1C,D). As is readily confirmed on bi-variate FACS plots similar to the one shown in Figure 1A, this increase is due to labeled cells having divided once and not new cells incorporating BrdU.

The kinetics of labeled cells early after the BrdU pulse are informative in several regards. They indicate that: (i) S and G2M phases lasts for about 5–6 h which is consistent with previous estimates for lymphocytes in vitro, (ii) many GC B cells survive and maintain a GC phenotype at least 5–6 h after their birth (otherwise the frequency in labeled cells could not increase by a factor close to two), and (iii) the overall turnover rate (cell entering cell cycle) in GCs is approximately 25% in 5–6 h (in line with 4% of BrdU+ EdU− cells per hour observed in recent measurements using the EdU/BrdU double pulse labeling approach [Supplementary Figure S3 in (54)]. A turnover rate of 4% per hour is remarkable: In steady state it implies that within 6 h, one in four GC B cells either undergoes apoptosis or leaves the GCs via differentiation and emigration (Figure 1D). Moreover, because of point (ii), many of these cells have not been in cell cycle recently.

A comparison of the relative increase in frequencies of BrdU+ cells in GC B and other LN cell population after the pulse shows a marked difference in kinetics (Figures 1E,F). While the level of BrdU+ cells among GC B cells quickly rises and then remains relatively stable at twofold of the initial value, frequencies of BrdU+ cells in the non-GC B cell populations steadily increase. This difference most likely reflects tight regulation and selection within GCs, as well as constant egress out of the GC. Indeed when BrdU+ cells in non-GC populations are analyzed for DNA content, they mostly appear in G0/G1 phase, therefore possibly representing differentiated GC emigrants which have downregulated GC expression markers and have stopped cycling (not shown).

### 3.2 GC B Cell’s CXCR4 Expression Increases Continuously Throughout Cell Cycle

**Figure 2:**
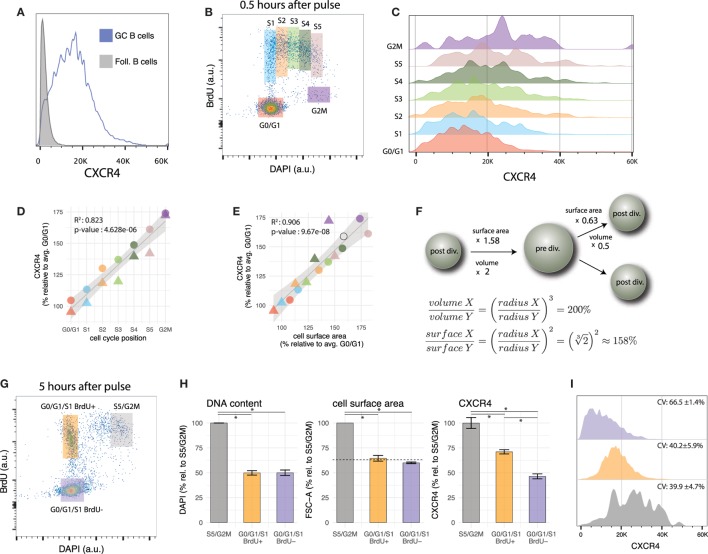
Cell cycle modulates CXCR4 expression in GC B cells. **(A)** CXCR4 expression in GC B cells is heterogeneous and its distribution is unimodal. CXCR4 expression profile of follicular B cells is shown as a negative reference. **(B)** Gates used to group GC B cells according to cell cycle position. **(C)** CXCR4 expression profile of GC B cells shifts to higher values as cells progress in cell cycle. **(D)** Average CXCR4 expression level increases with cell cycle position. Color and shape of the points indicate gate and mouse, respectively. **(E)** Average CXCR4 expression level increases linearly with cell surface area (as measured by forward scatter). The open circle indicates the relative increase in cell surface area expected from a doubling in volume of a perfect sphere. **(F)** Relative increase in surface area of a perfect sphere as it doubles/halves its volume, derived using calculus. **(G)** Representative example of bi-variate scatter plot of DAPI (DNA content) versus BrdU 5 h after BrdU pulse. Recently divided GC B cells are in G0/G1/S1 BrdU+ gate (orange), while cells that were in S phase during the pulse, and are about to divide are in the S5/G2M gate (gray). Relatively aged GC B cells that have completed their last division cycle more than 5 h ago (and rare cells that were in G2M phase during the pulse) are in the G0/G1/S1 BrdU− gate (violet). **(H)** DNA content (DAPI), cell surface area (FSC), and CXCR4 for the gates defined in panel **(G)**. The dashed line indicates the reduction in surface area of a perfect sphere that halves its volume. **(I)** CXCR4 expression profiles of cells in the S5/G2M, G0/G1/S1 BrdU+, G0/G1/S1 BrdU− gates. As cells divide, they pass from S5/G2M to the G0/G1 BrdU+ gate, resulting in a reduction of average CXCR4 expression by a factor of 0.71 ± 0.01 **(H)**, but no change in CV. The population with the highest CV (66.5 ± 1.4%) are the cells in the G0/G1/S1 BrdU− gate, having a low mean but a relatively large spread toward higher levels.

Despite mediating the segregation of GCs into two histologically well-defined zones (i.e., light zone and dark zone), CXCR4 expression profile in GC B cells is heterogeneous and does not show two distinguishable peaks (Figure 2A), as would be expected from a mixture of high expressing DZ (centroblasts) and low expressing LZ cells (centrocytes). This suggests that CXCR4 expression profiles of centroblasts and centrocytes cells overlap, a feature shared with many other genes expressed in DZ and LZ GC B cells (32). An exception (published in 2010 after the present data were generated (42)) is CD86, an accessory protein that plays a key role in T cell-B cell co-stimulation. Together with CXCR4, CD86 is currently the method of choice to gate LZ and DZ GC B cells by flow cytometry (e.g., Ref. (18, 54, 64–67)). Despite this important technical progress in terms of phenotypic discrimination of DZ and LZ cells, some questions regarding CXCR4 expression heterogeneity remain unanswered. For instance, do there exist two or more distinct CXCR4 expression levels in GC B cells that are blurred by stochastic noise or other sources of biological variability? And how is CXCR4 expression related to cell cycle and B cell receptor affinity?

In the present data, BrdU incorporation, DNA content and CXCR4 expression level have been recorded simultaneously with GC phenotypic markers. This permits determination of cell cycle position and CXCR4 expression level in GC B cells after the pulse. Grouping GC B cells into seven gates according to cell cycle position (G0/G1, S1 to S5 and G2M, mapped to cell cycle position {1, 2,…,7}, Figures 2B,C) reveals that CXCR4 expression steadily increases from G0/G1 to S and G2M phase (*R*^2^ = 0.82, *p*-value = 4.6*e*^–6^) with an average value in G2M reaching approximately 75% above G0/G1 levels (Figure 2D). When plotted against forward scatter, a proxy for cell surface area, CXCR4 also exhibits a linear relationship (Figure 2E, *R*^2^ = 0.90, *p-*value = 9.6*e*^–8^) with a slope not statistically different from 1 (*p*-value = 0.23). Together this argues for an increase in total numbers of CXCR4 receptors throughout cell cycle but maintenance of a relatively consistent surface density.

The change in cell surface area is a necessary consequence of the changes in volume of the cell that occur during cell cycle (68). While the relative increase/decrease depends on the precise shape of the cell, as a reference the increase in surface of a perfect sphere that doubles its volume is 58% (open circle in Figures 2E,F). When the sphere is split into two equally sized smaller spheres, the volume of each is halved, but surface areas are reduced by a factor of 0.63 (Figure 2F). As demonstrated in the next section, CXCR4 expression levels on GC B cells follow a similar trend.

### 3.3 Low CXCR4 Receptor Expression of GC B Cells in G0/G1/S1 That Have Not Been in Cell Cycle Recently

With CXCR4 expression increasing as cells approach mitosis, the next question that arises is what happens to the receptors when GC B cells divide. One can address this question (to a certain degree) by comparing, several hours after the BrdU pulse, CXCR4 expression of BrdU+ cells that have just divided, with those that are about to divide (Figure 2G). As anticipated from our previous results, this analysis shows that recently divided GC B cells in G0/G1/S1 (at this stage cells in early S phase cannot be distinguished from G0/G1 cells anymore) display in average lower numbers of CXCR4 receptors on their surface than their undivided peers in S5/G2M (Figure 2H). Several scenarios can be envisioned: for instance CXCR4 receptors are equally apportioned to the daughter cells (dilution), one of the daughter cells receives the majority of the receptors (asymmetric division), or CXCR4 receptor levels are continuously adjusted to cell’s surface area. A reduction by a factor 0.71 ± 0.01 not significantly different from the reduction in cell surface area by 0.64 ± 0.07 (*p*-value = 0.22) and almost identical coefficients of variation (CV) in CXCR4 expression between cells in S5/G2M and G0/G1/S1 BrdU+ (*p*-value for each mouse are {0.76, 0.96}, Figure 2I), argue against dilution (which would result in 50% reduction) or asymmetric apportioning (which would result in a higher CV) of the receptors to the two daughter cells, but suggest an actively regulated process, that maintains cell surface density of CXCR4 receptors approximately constant. Of note is that identical CVs for S5/G2M and G0/G1/S1 BrdU+ cells are in line with a scenario in which daughter cells inherit CXCR4 expression levels proportional to the mother cell (i.e., if a mother with relatively high/low CXCR4 expression and surface area generates daughter cells with relatively high/low CXCR4 expression and surface area).

When recently divided cells are compared to BrdU− cells in G0/G1/S1 phase (mostly cells or progeny of cells that are not and have not been in S phase in the last 5 h), the CXCR4 expression of the latter is significantly lower, although DNA content and surface area are not (Figure 2H, the dashed line indicates the reduction in surface area of a perfect sphere that halves its volume). The BrdU− G0/G1/S1 population is, therefore, likely to be enriched for relatively quiescent cells in the LZ, possibly undergoing selection or differentiation. While CXCR4 expression largely scales with cell surface area, additional factors may lead to a further downregulation in this population.

In summary, the above observations demonstrate a continuum of states in terms of CXCR4 expression levels between G0/G1 and M phase, and at least two distinct G0/G1/S1 GC B cell populations with intermediate and low CXCR4 expression levels that differ in their age (or time since last division) and probably location within the GC.

### 3.4 CXCR4 Expression Kinetics Are Different in Recently Divided and Relatively Aged GC B Cells as They Reenter Cell Cycle

In the previous section, two G1/G0/S1 GC B cell populations were identified that differ in terms of their average CXCR4 profile: recently divided BrdU+ with intermediate and relatively aged BrdU-negative cells with low expression levels, respectively. What remains unclear is how these two populations evolve over time and how they are related to each other. One possible scenario could be that some time after birth every cell further downregulates CXCR4 and migrates to the light zone, consistent with a model in which a selection step in the light zone occurs within each division cycle. Such a behavior would be reflected by a decrease in CXCR4 expression in the BrdU-positive G0/G1/S1 cell population, prior of G0/G1 cells entering S phase. Analysis of the present data indicates that this is not the case.

**Figure 3:**
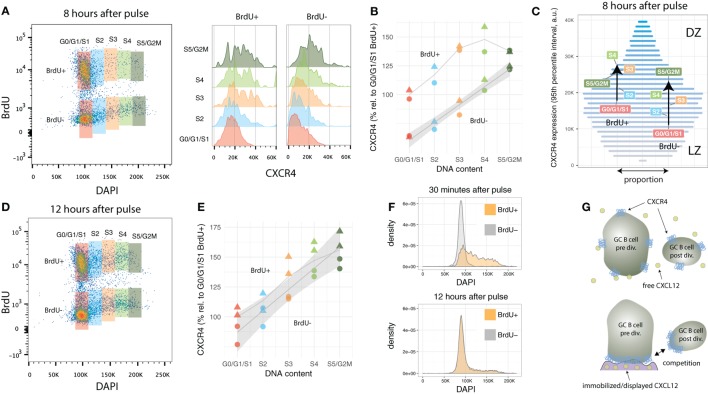
CXCR4 kinetics 8 and 12 h after BrdU pulse. **(A)** Bi-variate scatter plot of DAPI (DNA content) versus BrdU 8 h after the pulse and corresponding CXCR4 expression profiles. **(B)** Average CXCR4 expression increases with cell cycle position in both recently divided BrdU+ and relatively aged BrdU− cells. While BrdU+ cells reach a plateau in mid-S at about 50% above their original value, BrdU− cells’ increase in average CXCR4 expression is unabated until G2M phase. Color and shape of the points indicate gate and mouse, respectively. **(C)** Changes in average CXCR4 expression compared to a representative CXCR4 expression profile. Bar widths correspond to the proportion of GC B cells with a specific CXCR4 expression level (color and y-axis position). DZ and LZ labels indicate that high and low CXCR4 expression is typically attributed to DZ and LZ phenotype, respectively. **(D)** Bi-variate scatter plot of DAPI (DNA content) versus BrdU 12 h after the pulse. **(E)** CXCR4 expression in the BrdU+ and BrdU− subpopulation has converged indicating complete spatial mixing of individual GCs within approximately 12 h. Color and shape as in panel **(B)**. **(F)** Comparison of cell cycle distribution (DNA profile) of BrdU+ and BrdU− cells 30 min and 12 h after pulse. **(G)** Proposed theoretical model in which immobilized or displayed CXCL12 leads to competition between cells prior and post division due to their difference in total numbers of CXCR4 receptors. Crucial to this model is the polarization of CXCR4 to the cell’s leading edge in the presence of CXCL12, which has been reported for T cells and various tumor cell lines, but remains to be shown for GC B cells.

Both recently divided BrdU-positive and relatively aged BrdU-negative GC B cells in G0/G1/S1 maintain their CXCR4 expression levels. As the cells enter or progress in cell cycle, CXCR4 levels increase in both populations (Figures 3A,B). Recently divided cells reach a plateau in terms of average CXCR4 copy numbers in mid-S phase (at approximately 50% above G0/G1/S1 levels). By contrast, BrdU− cells, which had been in G0/G1 several hours prior of entering S phase, incessantly ramp up average expression, leading to a twofold increase in CXCR4 receptors at the end of the cell cycle (Figure 3B).

Figure 3C illustrates how the subpopulations defined in Figure 3A are positioned in terms of CXCR4 expression relative to the overall GC B cell population. The widths of the horizontal bars corresponds to a representative proportion of GC B cells with a given CXCR4 expression level, while their color (and y-axis position) is proportional to the expression level (low: gray, high: blue). For clarity, cells with extreme low and high CXCR4 expression outside the 95% percentiles were excluded from this analysis. In average, BrdU-negative G0/G1/S1 cells, as they enter and advance in cell cycle, traverse approximately one-third of the 95% expression interval largely “overtaking” recently divided cells in G0/G1/S1. The latter, however, when in mid-S phase reach slightly higher average values, but then stagnate and almost coincide with BrdU-negative cells in S5/G2M. With the current data, it was not possibly to distinguish whether the reduction in slope represents a general behavior (i.e., all recently divided GC B cells as they reenter cell cycle are following this trend) or whether some cells downregulate and other cells keep upregulating CXCR4 expression.

### 3.5 Desynchronization of CXCR4 Expression and Cell Cycle in BrdU+ GC B Cells 12 h After Pulse

Twelve hours after the BrdU pulse, CXCR4 levels in BrdU-positive are no longer distinguishable from BrdU-negative cells (Figures 3D,E). Similarly, DNA profiles of BrdU-positive and BrdU-negative cells are practically identical (Figure 3F). This is remarkable, as most clones underwent only one and maximally two divisions since the pulse. Such a rapid desynchronization is indicative for a high variability in GC B cell cycle progression speed, a phenomena perhaps linked to the selection process or diversity in affinity of hypermutated B cell receptors which has been shown to affect cell cycle speed (18).

## 4 Discussion

In this paper, published BrdU pulse-chase GC B cell data from draining lymph nodes 2 weeks after NP-CCG immunization is reanalyzed. Turnover rates (cells entering cell cycle) of 4% per hour are inferred for GCs B cells, not inconsistent with GC B cells dividing in average every 12 h. Despite this fast turnover, most newly divided cells are found to “survive” for over 5–6 h after their birth, a time in which hypermutated cells are likely to undergo selection required for affinity maturation.

The analysis further reveals, as its major finding, a so far unreported but potentially far-reaching relationship between GC B cell CXCR4 expression and cell cycle. Average numbers of CXCR4 receptors per cell scale linearly both with DNA content and forward scatter, a proxy for cell surface area. Compared to BrdU labeled cells in G2M, recently divided BrdU-positive GC B cells in G0/G1/S1 display 0.71 times less CXCR4 receptors on their surface. Expression is further reduced in BrdU-negative GC B cells in G0/G1/S1, which have not divided recently. Twelve hours after the pulse, BrdU labeled cells are indistinguishable from unlabeled cells both in terms of cell cycle and CXCR4 expression, suggesting a complete mixing of DZ and LZ in GCs within half a day. On a descriptive level, the analysis demonstrates a greater complexity in GC B cell CXCR4 expression than has previously been appreciated, by linking CXCR4’s heterogeneous expression profile to cell cycle progression and cell division.

What are the implications of the above observations on a theoretical model of the germinal center reaction. While it has long been known that cells in DZ and LZ differ in terms of cell cycle kinetics (28, 31, 63), the present analysis suggest that it is cell cycle progression and cell division itself that drives CXCR4 expression and therefore migration toward and against the CXCL12 gradient. The proposed model based on this (and other) data is as follows: as GC B cells progress through cell cycle, surface as well as CXCR4 expression increase concurrently. Assuming that CXCR4 in GC B cells polarizes on the cell’s leading edge in the presence of its ligand CXCL12, as has been reported for T cells and several CXCR4 expressing cancer cell lines (69, 70), higher absolute numbers of CXCR4 receptors entail an advantage to compete for space on CXCL12 presenting reticular cell networks (or immobilized CXCL12 on other surfaces) in the DZ (Figure 3G). When the mother cell divides, however, total CXCR4 expression levels in the two daughter cells drops, as does their surface area, and the offspring are no longer able to compete for CXCL12 binding. This leads to their displacement from the reticular cell network (or CXCL12 coated surfaces) by cells at later stages of the cell cycle with higher CXCR4 levels. As a result, the two daughter cells are being “pushed back” toward the LZ, consistent with the empirically determined net flux from DZ to LZ (3, 39, 40, 42). Some GC B cells reenter cell cycle rapidly and start increasing CXCR4 expression levels again (observed in the present data 8 h after the pulse), while others remain in G0/G1 and decrease CXCR4 further (deduced from the observation that labeled and non-labeled cells mix within 12 h). A proportion of cells with low CXCR4 expression levels that dwell in G0/G1 phase for several hours, reenter S phase, to reach CXCR4 expression levels in G2M similar to cells that reenter S phase immediately after their birth. CXCR4 low expressors in G0/G1 that do not reenter cell cycle are prone to leave the GC (low levels of CXCR4 could let these cells “escape” the CXCR12 gradient) as memory or plasma cells or undergo apoptosis, while intermediate expressors are expanding clones that do not require T cell help for a further rounds of division.

The data analyzed here shows a strong correlation between cell cycle and CXCR4 expression. The proposed model assumes that it is cell cycle that drives CXCR4, but one could argue that it may as well be CXCR4 that regulates cell cycle instead. Evidence that this is most likely not the case comes from a CXCR4 knockout study (26) (similar results have been reported for Foxo1 knockouts (64–66)), which demonstrates that the lack of expression of this gene does not have a major effect on the magnitude of the GC reaction nor the proportions of cells with light and dark zone phenotype. Intriguingly, however, in contrast to proliferation and differentiation, affinity maturation is impaired in these mice. Thus, it seems that GC B cell depend on the CXCR4/CXCL12 axis for effective selection of higher affinity hypermutated variants. How could this relate to cell cycle modulation of CXCR4 levels? Perhaps higher CXCR4 expression levels in G2M ensure that crucial processes like BCR signaling in G2 (71), cytokinesis and AID activity in early G1 phase (72) happen preferentially in proximity to CXCL12, either on the expressing reticular cell network (8) or CXCL12 immobilized on other surfaces (67). On the other hand, high CXCR4 expression could also enhance BCR signaling in G2 phase, as has been described for the T cell receptor (73), perhaps relying on the recently discovered colocalization of CXCR4, BCR immunoglobulin D, and CD19, in mature B cells (74). Finally increased CXCR4–CXCL12 interaction strength could also potentially facilitate asymmetric cell division (75) by inducing polarization (as reported during T cell development (76)), although in the present data there was no evidence for asymmetric apportioning of CXCR4 to daughter cells. Further studies are needed to answer these questions.

Similar to the CXCR4 knockout, it was shown that for CXCL12*^gagtm^* mice, in which CXCL12 is unable to bind cellular or extra-cellular surfaces, magnitude of the germinal center reactions is normal but affinity maturation is less effective (67). Two observations reported in this study are particularly relevant here. A first one is that GC B cross-section cell surface areas are heterogeneous but significantly larger in DZ then in LZ. A second one is that CXCL12*^gagtm^* GC B cells in G2M phase are found almost as frequently in LZ as in DZ while in wild-type controls the majority is found in the DZ only. Both observations are in line with the model proposed above in which a CXCL12 gradient serves as a guide for cycling cells to reach CXCL12 high regions when approaching G2M phase.

The weakness (and perhaps strength) of this work is the small number of samples it is based on (i.e., 10 mice in total) and the fact that the data were created using a single experimental technique. Clearly the hypotheses generated by this study remain to be challenged in future experiments. Repeats with different immunization protocols, timings, and mouse strains will help to test the robustness of the observed relationships and kinetics. And additional markers, for instance Ki-67 to separate G0 and G1 cells, a second EdU pulse at later time points to distinguish S1 from G0/G1 (54), Blimp-1 to identify plasma blasts (16), and/or the recently discovered marker Ephrin-B1 which marks mature GC B cells (77), will aid to further resolve the fate, cell cycle, and CXCR4 expression levels of relevant subpopulations. Technically more advanced approaches, for instance continuously monitoring CXCR4 expression in cycling GC B cells from CXCR4 cross FUCCI reporter mice, via in vitro long-term imaging and tracking would certainly be highly informative (78, 79), as would be GC B single-cell RNA sequencing experiments (80).

Beyond its function in affinity maturation, CXCR4 is implicated in regulating numerous other vital processes, for example, embryonic development (81, 82), hematopoietic stem cell self-renewal in the bone marrow (83), and neutrophil release during stress (84). Its role in disease further highlights its relevance in cellular homing and proliferation. CXCR4 is overexpressed in more than 23 human cancers (85) including leukemia (86), is associated with metastasation (87), and has been identified as a marker for poor prognosis in human patients (88). For HIV it represents a major co-factor for entry into T-cells during the immunological deficient phase of infection (89). If cell cycle modulates CXCR4 expression in GC B cell, as the data analyzed here indicates, it will be important to investigate whether this mechanism is specific to GCs or whether it also plays a role in other tissues and cell types.

## Author Contributions

TW performed the analysis, developed the hypotheses, and wrote the manuscript.

## Conflict of Interest Statement

The author declares that the research was conducted in the absence of any commercial or financial relationships that could be construed as a potential conflict of interest.
