# Supplementary material for: Cell Cycle-Associated CXCR4 Expression in Germinal Center B Cells and Its Implications on Affinity Maturation
Source: Front Immunol. 2018 Jun 12;9:1313. doi: 10.3389/fimmu.2018.01313 (PMC6008520; doi:10.3389/fimmu.2018.01313)
Supplement: Supplementary file 1 [file image_1.PDF]

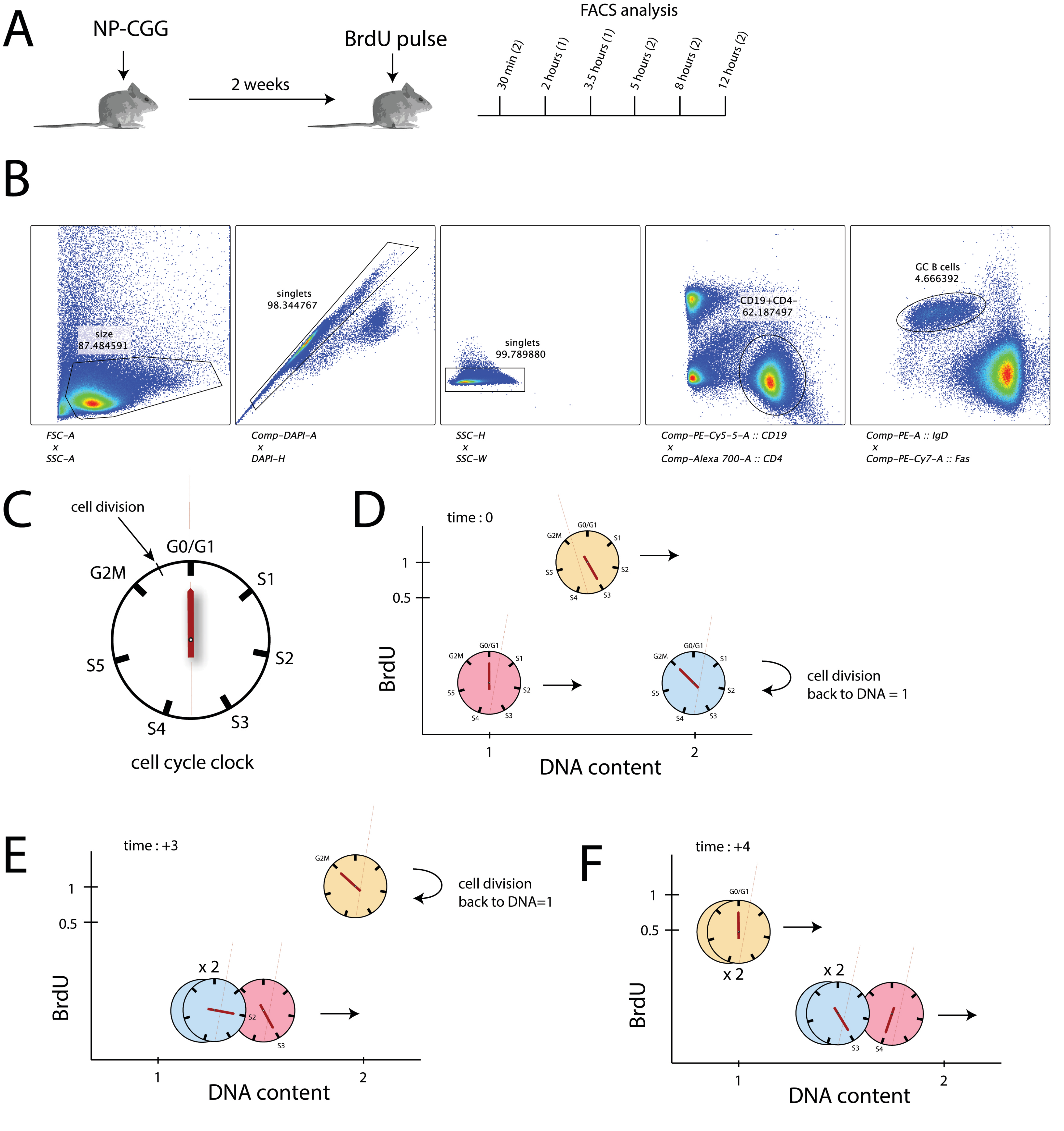

Figure S1. Experimental setup and interpretation of BrdU pulse-chase data. A) Overview of immunization and BrdU labeling protocol. Numbers in brackets give the number of mice sampled at each time point. **B)** Gating strategy used to isolate single GC B cells. **C)** Simplified cell cycle clock model with G0/G1, S1, S2, S3, S4, S5, and G2M as discrete phases. For the purpose of illustration all phases are assumed to have the same duration (1 hour) and cell division occurs between G2M and G1. **D)** Three hypothetical 'cells' in different phases of the cell cycle immediately after a BrdU pulse. Cell cycle position determines position of the DNA versus BrdU plot. As cells continue replication they 'move' to the right as DNA content increases, eventually divide, and 'jump back' to DNA content 1 at division. **E)** Three hours after the pulse, the BrdU labelled cell in panel D has reached G2M phase, the cell initially in G0/G1 is half-way through S phase, and the cell initially in G2M has divided and its progeny are in early S phase. **F)** Four hours after the pulse, the BrdU+ cell has divided as well, and the three unlabeled cells have moved further to the right.
